# Overcoming drug-tolerant cancer cell subpopulations showing AXL activation and epithelial–mesenchymal transition is critical in conquering ALK-positive lung cancer

**DOI:** 10.18632/oncotarget.25531

**Published:** 2018-06-05

**Authors:** Shinji Nakamichi, Masahiro Seike, Akihiko Miyanaga, Mika Chiba, Fenfei Zou, Akiko Takahashi, Arimi Ishikawa, Shinobu Kunugi, Rintaro Noro, Kaoru Kubota, Akihiko Gemma

**Affiliations:** ^1^ Department of Pulmonary Medicine and Oncology, Graduate School of Medicine, Nippon Medical School, Tokyo 113-8603, Japan; ^2^ Department of Analytic Human Pathology, Graduate School of Medicine, Nippon Medical School, Tokyo 113-8603, Japan

**Keywords:** ALK, lung cancer, resistance, EMT, AXL

## Abstract

Anaplastic lymphoma kinase tyrosine kinase inhibitors (ALK-TKIs) induce a dramatic response in non–small cell lung cancer (NSCLC) patients with the *ALK* fusion gene. However, acquired resistance to ALK-TKIs remains an inevitable problem. In this study, we aimed to discover novel therapeutic targets to conquer ALK-positive lung cancer. We established three types of ALK-TKI (crizotinib, alectinib and ceritinib)-resistant H2228 NSCLC cell lines by high exposure and stepwise methods. We found these cells showed a loss of ALK signaling, overexpressed AXL with epithelial-mesenchymal transition (EMT), and had cancer stem cell-like (CSC) properties, suggesting drug-tolerant cancer cell subpopulations. Similarly, we demonstrated that TGF-β1 treated H2228 cells also showed AXL overexpression with EMT features and ALK-TKI resistance. The AXL inhibitor, R428, or HSP90 inhibitor, ganetespib, were effective in reversing ALK-TKI resistance and EMT changes in both ALK-TKI-resistant and TGF-β1-exposed H2228 cells. Tumor volumes of xenograft mice implanted with established H2228-ceritinib-resistant (H2228-CER) cells were significantly reduced after treatment with ganetespib, or ganetespib in combination with ceritinib. Some ALK-positive NSCLC patients with AXL overexpression showed a poorer response to crizotinib therapy than patients with a low expression of AXL. ALK signaling-independent AXL overexpressed in drug-tolerant cancer cell subpopulations with EMT and CSC features may be commonly involved commonly involved in intrinsic and acquired resistance to ALK-TKIs. This suggests AXL and HSP90 inhibitors may be promising therapeutic drugs to overcome drug-tolerant cancer cell subpopulations in ALK-positive NSCLC patients for the reason that ALK-positive NSCLC cells do not live through ALK-TKI therapy.

## INTRODUCTION

The anaplastic lymphoma kinase (*ALK*) fusion gene is found in about 3-5% of patients with non-small cell lung cancer (NSCLC) [1, 2]. *ALK* fusion gene–positive NSCLC patients showed a dramatic response to ALK tyrosine kinase inhibitors (ALK-TKIs) such as the first generation ALK-TKI, crizotinib, and second generation ALK-TKIs, alectinib and ceritinib [3–5]. However, acquired resistance to ALK-TKIs remains a virtually inevitable issue.

Two major mechanisms of resistance to crizotinib in *ALK*-positive NSCLC patients have been previously described. ALK signal-dependent activation, such as ALK secondary mutations, and copy number amplification, have been reported in about half of resistant tumors [6–10]. ALK-positive NSCLC tumors showed multiple gatekeeper mutations, including 1151Tins, L1152R, C1156Y, F1174L, L1196M, G1202R, S1206Y and G1269A, after treatment with crizotinib. Another crizotinib-resistant mechanism is the activation of alternative survival signaling pathways, including EGFR activation, KIT activation having a *KRAS* mutation and IGF-1R activation [6, 7, 9–11]. The activation of bypass pathways has also been found to be a mechanism of resistance to alectinib and ceritinib [12–14]. Alternative signaling activation, such as MET against crizotinib, RET against alectinib and IGF-1R and INSR against ceritinib, has also been reported [10, 15, 16]. However, the development of drug resistance in NSCLC patients with *ALK* is a major challenge that needs to be overcome.

In this study, we established three types of ALK-TKI-resistant NSCLC cell lines (crizotinib-resistant H2228-CRR cells, alectinib-resistant H2228-ALR cells and ceritinib-resistant H2228-CER cells) from a H2228 cell line harboring *EML4-ALK*. These ALK-TKI–resistant cells commonly lost the *EML4*-*ALK* driver oncogene. The purpose of this study was to establish novel therapeutic strategies to eradicate cancer cells in ALK-positive NSCLC patients.

## RESULTS

### Establishment of ALK-TKI–resistant H2228 cell lines by high exposure and stepwise methods

**Figure 1:**
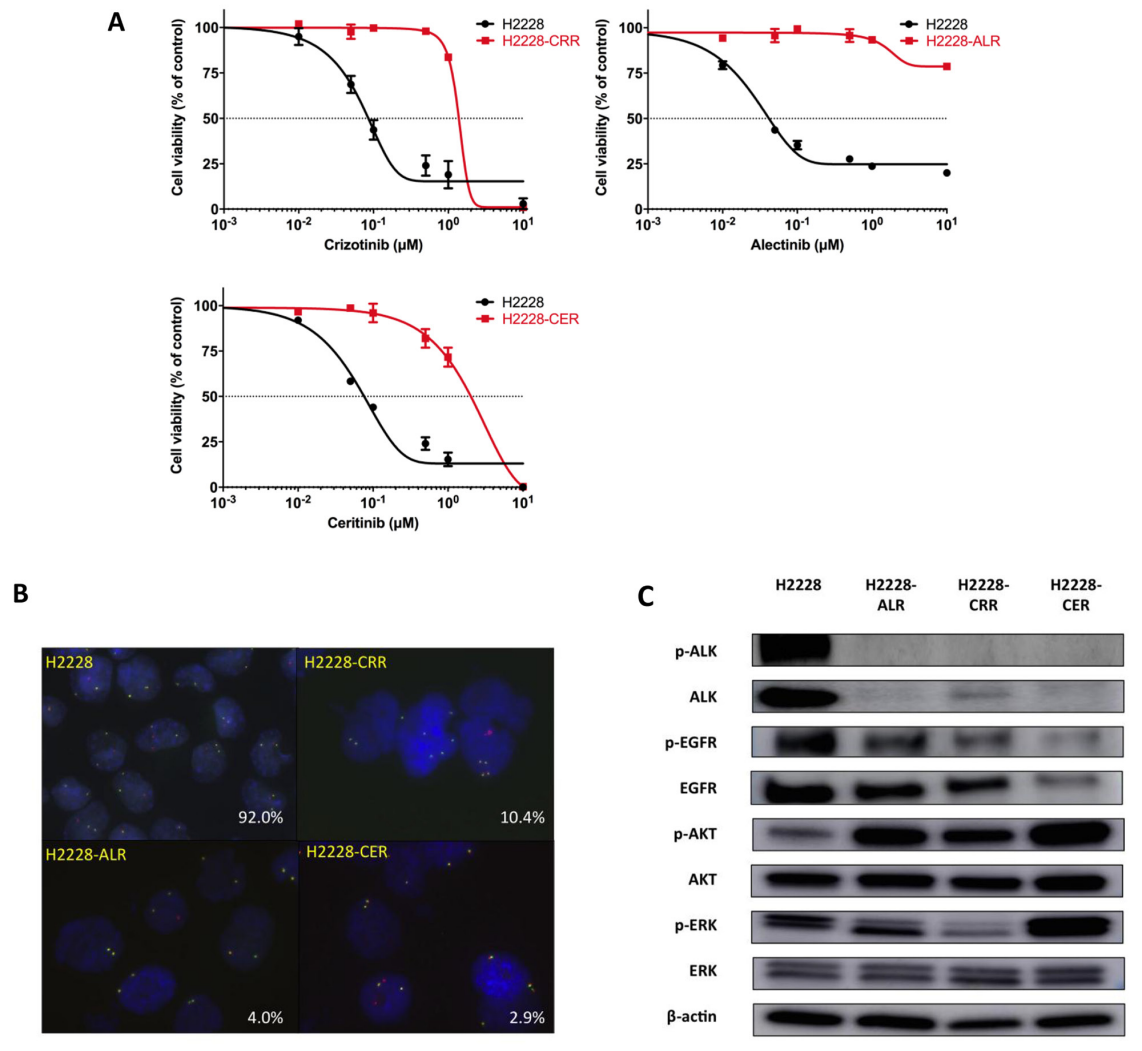
Establishment of ALK-TKI–resistant H2228 cells **(A)** H2228-CRR, H2228-ALR and H2228-CER cells are resistant to crizotinib, alectinib and ceritinib, respectively. The results of cell viability assays are shown. Data, mean ± SEM from three independent experiments. **(B)** FISH analysis shows a decrease of the *ALK* fusion gene in ALK-TKI–resistant H2228 cells compared with H2228 cells (red, *ALK3’*; green, *ALK5’*). The percentage of ALK-FISH positive cells are shown in the lower right corners. **(C)** Protein levels of p-ALK, ALK, p-EGFR, EGFR, p-AKT, AKT, p-ERK and ERK were analyzed by western blotting. ALK-TKI–resistant cells showed markedly decreased p-ALK and ALK expressions.

We first evaluated the antitumor effects of crizotinib, alectinib, and ceritinib in H2228 cells by cell viability assay. H2228 cells were sensitive to all ALK-TKIs. Based on the 50% inhibitory concentration (IC_50_) of each ALK-TKI, we next established crizotinib-resistant (H2228-CRR), alectinib-resistant (H2228-ALR), and ceritinib-resistant (H2228-CER) H2228 cell lines by combining both high exposure and stepwise methods over a period of one year. We exposed H2228 cells to a high concentration of drugs (1 μM) and carefully cultured the few surviving cells in the absence of drugs. When the surviving cells gradually grew, we exposed these to a 1.5 times higher concentration of drugs (1.5 μM). By repeating these methods, we generated resistant cells. H2228-CRR, H2228-ALR and H2228-CER survived in concentrations of up to 3 μM crizotinib, 5 μM alectinib, and 2 μM ceritinib, respectively. IC_50_ values of crizotinib for H2228-CRR cells, alectinib for H2228-ALR cells and ceritinib for H2228-CER cells were 1.36, ≥ 10, and 1.55 μM, respectively; these cells were 16-fold, 233-fold or more, and 19-fold more resistant, respectively, than parental H2228 cells (Table 1 and Figure 1A). The IC_50_ values for each ALK-TKI in established ALK-TKI resistant cell lines in the absence of the ALK-TKI was still at quite a high concentration after a month. These resistant cell lines showed cross resistance to the other ALK-TKIs (Table 1). We confirmed that such resistant cells were derived from the parental cells using PCR analysis of short tandem repeats by a PowerPlex^®^ 16 STR System (Cell Authentication Report: KBN0275; JCRB Cell Bank, Osaka, Japan).

**Table 1:** IC_50_ values in parental and established ALK-TKI–resistant H2228 cells

	Crizotinib		Alectinib		Ceritinib	
	IC_50_ (μM)	Fold change	IC_50_ (μM)	Fold change	IC_50_ (μM)	Fold change
H2228	0.086	−	0.043	−	0.080	−
H2228-CRR	1.36	16	>10	>233	1.36	17
H2228-ALR	1.16	13	>10	>233	0.93	12
H2228-CER	1.42	17	>10	>233	1.55	19

All experiments were performed three times.

Abbreviations: ALK-TKI, anaplastic lymphoma kinase tyrosine kinase inhibitors; IC_50_, inhibitory concentration showing a 50% response.

We carried out exome sequence analysis using DNA from parental and ALK-TKI–resistant cells on an Illumina HiSeq 2500 platform using paired-end reads. ALK secondary mutations that had previously been reported to activate ALK, such as 1151Tins, L1152R, C1156Y, F1174L, L1196M, G1202R, S1206Y, and G1269A, were not observed in ALK-TKI-resistant cells (data are not shown). We also evaluated the *ALK* gene status of ALK-TKI-resistant cells. FISH analysis showed a decrease of the *ALK* fusion gene in ALK-TKI-resistant compared with parental H2228 cells. ALK translocation by FISH analysis was detected in 92.0% of H2228 cells, 10.4% of H2228-CRR cells, 4.0% of H2228-ALR cells, and 2.9% of H2228-CER cells for each 1000 cells (Figure 1B). Markedly decreased levels of p-ALK and ALK protein expression were also observed in ALK-TKI–resistant cells by western blotting (Figure 1C). Therefore, such ALK-TKI–resistant NSCLC cells survived independently of an ALK signaling pathway.

### AXL overexpression with EMT changes in ALK-TKI–resistant H2228 cells

**Figure 2:**
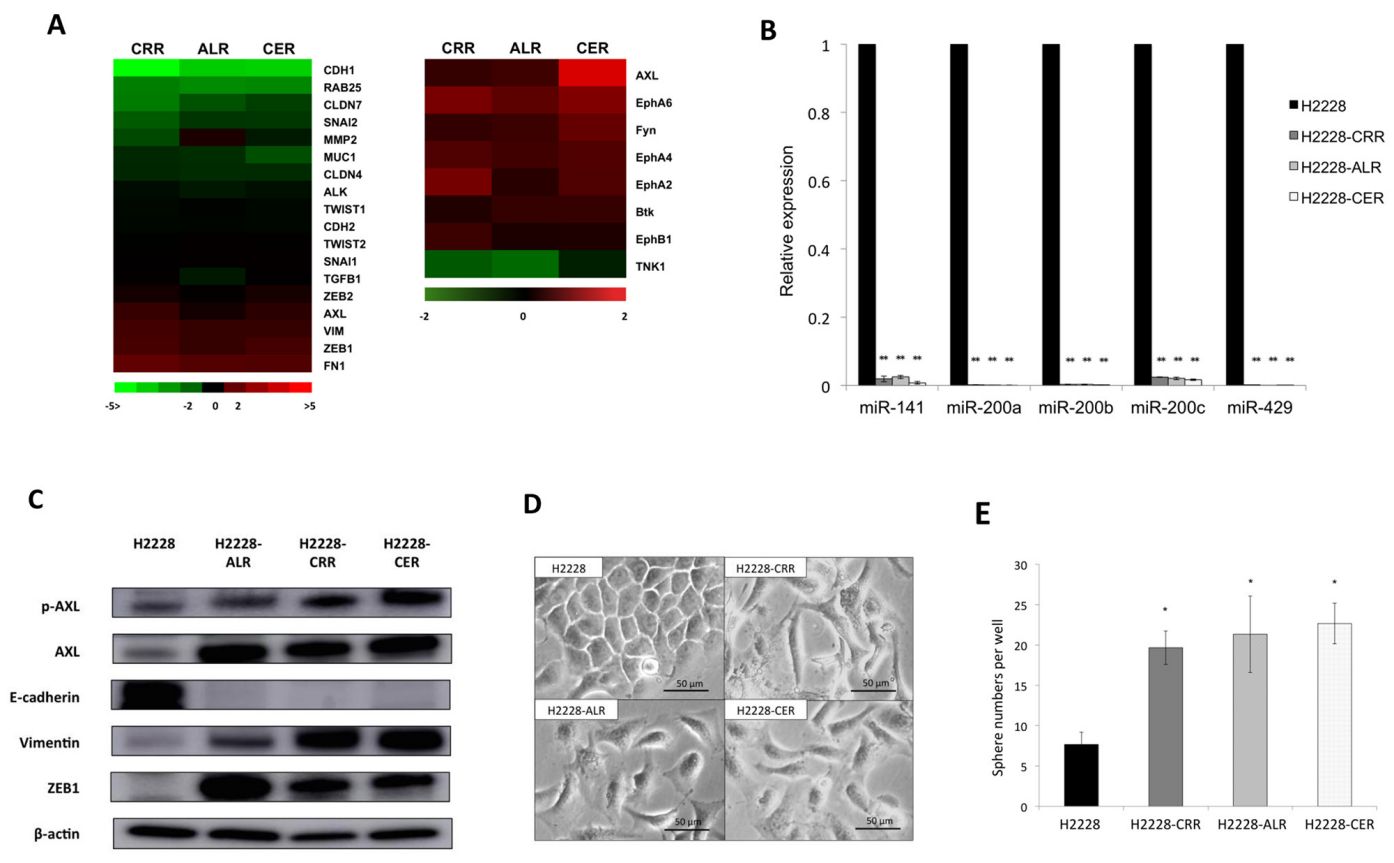
AXL overexpression with EMT features in ALK-TKI–resistant cells **(A)** The cDNA microarrays undertaken in ALK-TKI–resistant cells compared with parental cells are shown by an *ALK*- and EMT-related gene heat map using a log_2_ ratio [log_2_(ALK-TKI–resistant H2228 cells/H2228 cells)](left panel). *AXL*, *VIM*, *ZEB1* and *FN1* were overexpressed and *CDH1* was suppressed. Receptor tyrosine kinase (RTK) phosphorylation antibody arrays in ALK-TKI–resistant cells compared with parental cells are shown by a heat map using a log_2_ ratio [log_2_(ALK-TKI–resistant H2228 cells/H2228 cells)] with common log_2_ ratio changes of >2 or <2) (right panel). AXL was commonly upregulated in ALK-TKI–resistant H2228 cells. **(B)** The expression of miR-200 family members was suppressed in ALK-TKI–resistant compared with parental H2228 cells as determined by quantitative real-time reverse transcription PCR (qRT-PCR; ^**^p < 0.01). **(C)** Protein levels of AXL and epithelial–mesenchymal transition (EMT) markers were analyzed by western blotting. Increased p-AXL, AXL, vimentin and ZEB1 levels and a decreased E-cadherin level were observed in ALK-TKI–resistant H2228 cells. **(D)** Morphologic changes are observed under a light microscope in parental and ALK-TKI–resistant H2228 cells. H2228-CRR, H2228-ALR and H2228-CER cells showed mesenchymal features. **(E)** The numbers of spheres increased in ALK-TKI–resistant H2228 cells compared with parental H2228 cells in the sphere formation assay (^*^p < 0.05).

To identify common genes associated with resistance to ALK-TKIs in ALK-TKI–resistant cells, gene expression profiles were examined in parental and ALK-TKI–resistant H2228 cells by cDNA microarrays (Supplementary Figure 1A). *CDH1* encoding E-cadherin was the most downregulated gene in H2228-CRR compared to H2228 cells among 38,654 genes. *CDH1* gene expression was also strongly downregulated in H2228-ALR and H2228-CER cells. Because the low expression of E-cadherin suggested the occurrence of an epithelial-mesenchymal transition (EMT) phenotype [17], we evaluated the expression of other EMT-related genes: *FN1*, *ZEB1*, *VIM* encoding vimentin, and *AXL* were overexpressed in ALK-TKI-resistant cells (Figure 2A). *ALK* gene expression was decreased in all ALK-TKI-resistant cells. RTK phosphorylation expression profiles were also investigated in order to identify tyrosine kinase receptors in parental H2228 and ALK-TKI-resistant H2228 cells. AXL kinase was significantly upregulated in three ALK-TKI–resistant cell lines compared to parental cells. AXL was the most commonly upregulated kinase in H2228-CER compared to H2228 cells among 71 kinases. AXL was also a strongly upregulated kinase in H2228-CRR and H2228-ALR cells (Figure 2A).

We next evaluated miRNA expression levels by miRNA arrays. By targeting *ZEB1* and *ZEB2*, several members of the miR-200 family (miR-141, miR-200a, miR-200b, miR-200c and miR-429) are considered the main suppressors of EMT. The expressions of such miR-200 family members were all markedly suppressed in ALK-TKI-resistant compared with parental H2228 cells (data are not shown). We confirmed significant downregulation of miR-141, miR-200a, miR-200b, miR-200c and miR-429 in ALK-TKI-resistant cells as determined by qRT-PCR (Figure 2B). Upregulated AXL was found in both mRNA and protein phosphorylation expression profiles. We found increased protein levels of p-AXL, AXL, vimentin and ZEB1, and decreased E-cadherin protein in ALK-TKI-resistant cells by western blotting analysis (Figure 2C). To confirm the robustness of upregulated AXL in ALK-TKI-resistant H2228 cells, cloned ALK-TKI–resistant H2228 cells derived from a single cell by limiting dilution method were generated. Two independent clones of each ALK-TKI-resistant H2228 cell line also showed increased protein levels of p-AXL and AXL by western blotting analysis (Supplementary Figure 1B).

Morphologic changes from epithelial to spindle-type mesenchymal cells were observed in ALK-TKI-resistant H2228 cells compared with parental H2228 cells (Figure 2D), indicating resistant cells showed an EMT phenotype. These data suggest that AXL was responsible for the EMT phenotype and that these cells showed ALK-signaling independent resistance.

We also examined whether cancer stem cell-like (CSC) properties existed in such ALK-TKI-resistant cells, which are closely linked to EMT features [18]. Increased sphere number and size were found in ALK-TKI-resistant H2228 cells by sphere formation assay (Figure 2E and Supplementary Figure 1C). Such ALK-TKI-resistant cells may acquire CSC properties as well as EMT features.

### Transforming growth factor-β1-induced AXL overexpression and EMT changes in H2228 cells

**Figure 3:**
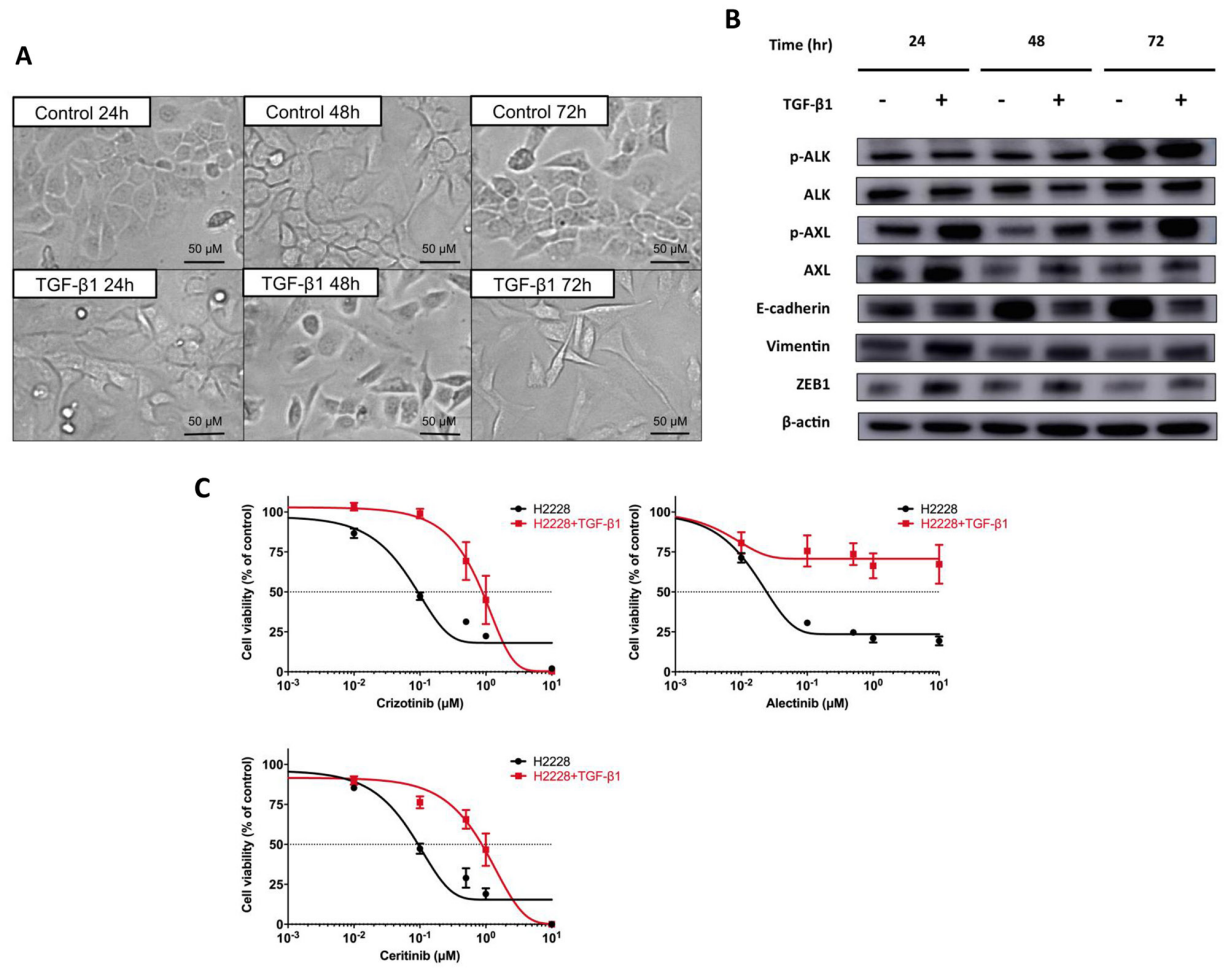
EMT changes and resistance to ALK-TKIs in H2228 cells exposed to TGF-β1 **(A)** Morphologic changes showing mesenchymal features in H2228 cells were observed after exposure of cells to 5 ng/mL TGF-β1. **(B)** Protein expression levels of ALK, AXL and EMT markers, after exposure of 5 ng/mL TGF-β1 for 6 hours to H2228 cells, were examined by western blotting. The expression of p-AXL, AXL, vimentin and ZEB1 increased in a time-dependent manner. **(C)** Resistance to crizotinib, alectinib and ceritinib was caused by exposure of H2228 cells to 5 ng/mL TGF-β1. The results of cell viability assays are shown.

To further confirm the correlation between AXL upregulation and an EMT phenotype, H2228 cells were stimulated by transforming growth factor β1 (TGF-β1). Previous studies had demonstrated that AXL expression and EMT in NSCLC cells could be induced by treatment with TGF-β1 [19, 20]. Figure 3A shows that changes from epithelial to mesenchymal cells, characterized by a spindle-type cell morphology, were observed in H2228 cells in a time-dependent manner after 5 ng/mL TGF-β1 treatment for 6 hours. Increased protein levels of p-AXL, AXL, vimentin and ZEB1, and decreased levels of E-cadherin were found in H2228 cells treated with 5 ng/mL TGF-β1 for 6 hours (Figure 3B). Expression levels of p-ALK and ALK were unchanged after TGF-β1 stimulation. Furthermore, resistance to crizotinib, alectinib and ceritinib were caused by 5 ng/mL TGF-β1 exposure for 6 hours in H2228 cells as determined by cell viability assay (Figure 3C). IC_50_ values of TGF-β1-exposed H2228 cells for crizotinib, alectinib, and ceritinib were 0.88, ≥ 10 and 0.89 μM, respectively, which were 10-fold, 192-fold or more, and 9-fold more resistant, respectively, than H2228 cells. These findings suggested that AXL activation and EMT were commonly involved in the resistance to ALK-TKIs in ALK-positive NSCLC cells, with or without ALK signaling.

### AXL and HSP90 inhibitors reverse EMT changes and overcome resistance

We next evaluated whether AXL inhibition reversed EMT and whether ALK-TKI-resistant cells responded to these inhibitors. We also screened chemical compounds to identify a candidate drug for ALK-TKI-resistant cells using a SCADS Inhibitor Kit III, which included 95 chemical compounds that each inhibited a specific molecular target, as previously described [21]. We focused on the HSP90 inhibitor, radicicol, because of its common effect on H2228 cells and three ALK-TKI-resistant cell lines. A previous study reported that a HSP90 inhibitor could overcome cellular resistance to an ALK inhibitor [22]. In H2228, H2228-CRR, H2228-ALR and H2228-CER cells, radicicol at 500 nM induced a 38%, 14%, 41% and 11% reduction of cell viability after 72 hours compared with a control inhibitor, respectively (Supplementary Table 1). Radicicol is a first generation HSP90 inhibitor; however, we also assessed a second generation HSP90 inhibitor, ganetespib, as this has previously shown promising data in a phase II trial conducted in NSCLC patients [23, 24]. Thus, we performed further experiments using a commercially available AXL inhibitor R428 and ganetespib as well as AXL siRNA to evaluate the reversal of EMT and their effectiveness in ALK-TKI-resistant cells.

**Figure 4:**
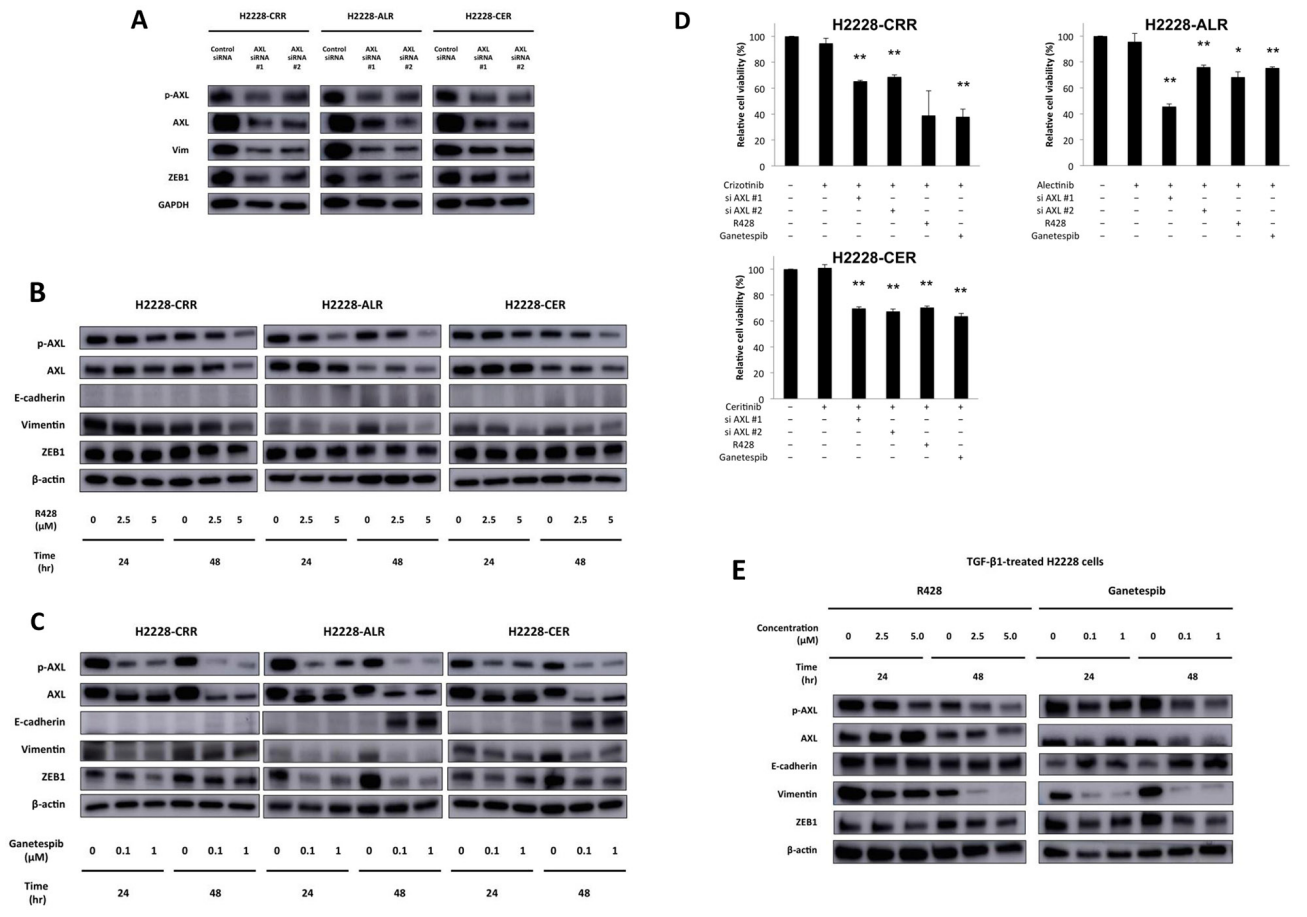
Reversal of EMT and ALK-TKI–resistance by R428 and ganetespib in ALK-TKI–resistant H2228 cells and H2228 cells exposed to TGF-β1 **(A)** Western blot analysis showed AXL knockdown with AXL siRNA caused the reversal of EMT markers in ALK-TKI–resistant H2228 cells. **(B, C)** Protein expression of AXL and EMT markers, as shown by western blot, in ALK-TKI–resistant cells after treatment with R428, an AXL inhibitor, and ganetespib, an HSP90 inhibitor, are shown in a time- and dose-dependent manner. R428 and ganetespib reversed EMT changes in ALK-TKI–resistant cells. **(D)** Cell viabilities were significantly reduced in ALK-TKI–resistant cells that had decreased ALK levels in response to AXL siRNA, R428 or ganetespib treatments. Crizotinib, alectinib and ceritinib were used at 100 nM. R428 and ganetespib was used at 2.5 μM and 100 nM, respectively (^**^p < 0.01 and ^*^p < 0.05). **(E)** Protein expression of ALK, AXL and EMT markers, as shown by western blot, in TGF-β1 treated H2228 cells after R428 and ganetespib treatment are shown in a time- and dose-dependent manner. R428 and ganetespib reversed EMT changes in TGF-β1 treated H2228 cells.

We found that AXL knockdown with AXL siRNA caused the reversal of EMT markers, including ZEB1 and vimentin, in ALK-TKI-resistant H2228 cells (Figure 4A). We also evaluated protein expression levels of AXL and EMT markers after R428 or ganetespib treatments of ALK-TKI-resistant H2228 cells. In both Figure 4B and 4C, AXL and p-AXL protein expression by western blot analysis decreased in a time-and concentration-dependent manner. R428 successfully reduced vimentin, as well as AXL and p-AXL expression (Figure 4B). Ganetespib successfully inhibited vimentin and ZEB1, as well as AXL and p-AXL expression. E-cadherin expression was observed after ganetespib treatment for 48 hours in H2228-ALR and H2228-CER cells (Figure 4C). R428 and ganetespib reversed EMT changes in all ALK-TKI-resistant cells in a dose-and time-dependent manner. Cell viabilities were significantly reduced in ALK-TKI-resistant cells that had decreased ALK levels in response to AXL siRNA, R428 or ganetespib treatments (Figure 4D). Furthermore, a decrease in p-AXL, resulting in a reduction of vimentin and ZEB1, was found in TGF-β1-treated H2228 cells after R428 treatment (Figure 4E). Inhibition of p-AXL, AXL, vimentin and ZEB1, and the induction of E-cadherin were observed in TGF-β1 treated H2228 cells after ganetespib treatment (Figure 4E). The disappearance of mesenchymal features in ALK-TKI-resistant cell lines was observed 48 hours after the treatment of cells with 2.5 μM R428 or 100 nM ganetespib (Supplementary Figure 2A). R428 or ganetespib were more effective in TGF-β1 exposed H2228 cells than in parental H2228 cells (Supplementary Figure 2B). IC_50_ values of TGF-β1 exposed H2228 cells against R428 and ganetespib were 0.68 μM and 0.014 μM, which were 10-fold and 714-fold or more effective than H2228 cells. Furthermore, resistance to ALK-TKIs stimulated by TGF-β1 could be restored by treatment with R428 or ganetespib (Supplementary Figure 2C). Taken together, these findings indicate that AXL and HSP 90 inhibitors targeting AXL may be promising agents for both ALK signal-independent and ALK-positive and TGF-β1-treated H2228 cells with ALK-TKI resistance.

### *In vivo* tumor experiments and correlation between AXL expression and crizotinib sensitivity in clinical tissue samples

**Figure 5:**
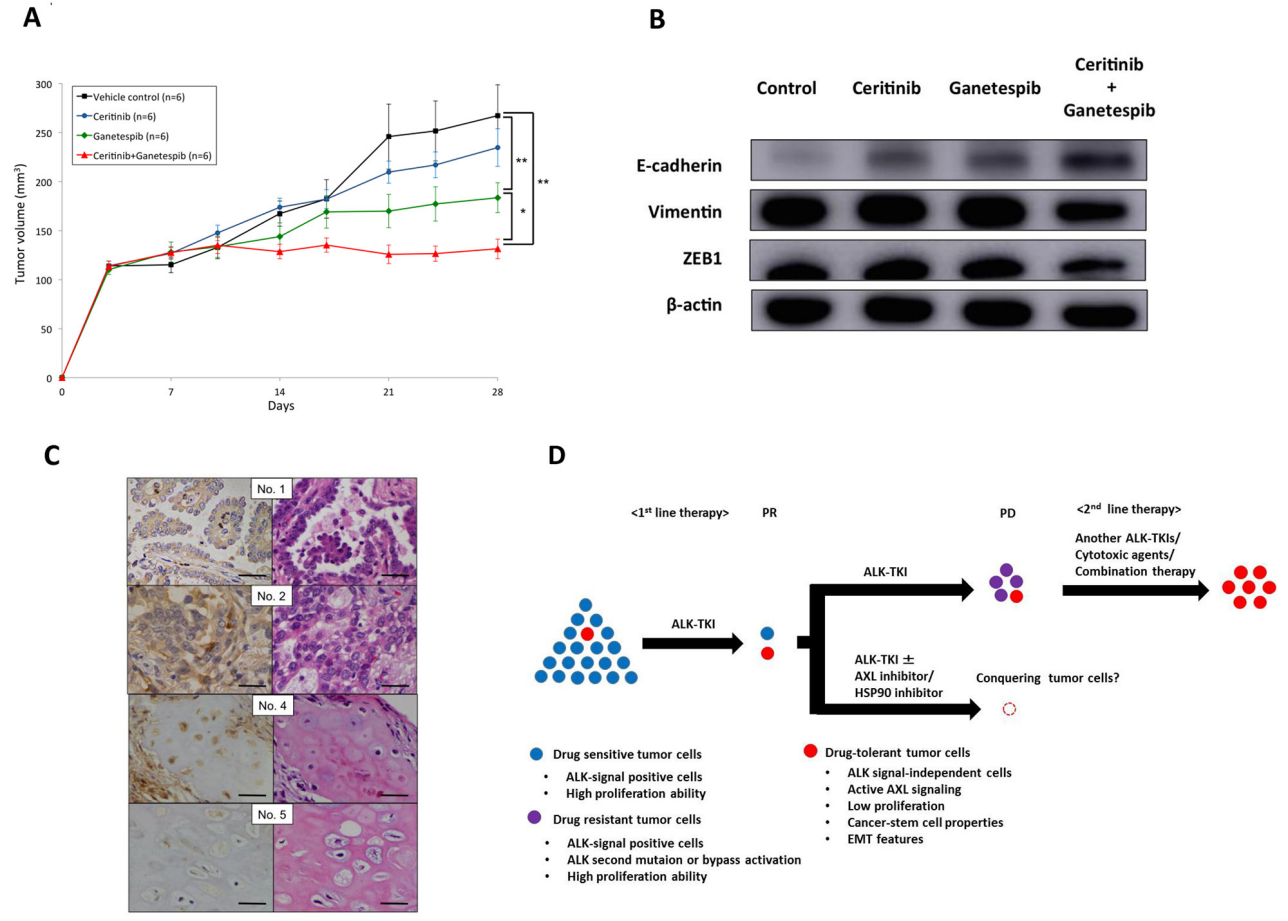
Significance of EMT changes in an *in vivo* xenograft mouse model and in ALK-fusion–positive patients **(A)** Tumor volumes changes in response to the administration of vehicle control, ceritinib, ganetespib or ganetespib in combination with ceritinib in a H2228-CER cell xenograft mouse model are shown. Ganetespib or ganetespib in combination with ceritinib were more effective than control vehicle and ceritinib treatment (^**^p < 0.01). **(B)** Protein expression levels of EMT markers after 28 days treatment with vehicle control, ceritinib, ganetespib or ganetespib in combination with ceritinib in a H2228-CER cell xenograft mouse model are shown by western blot analysis. **(C)** AXL expression in ALK-positive NSCLC patients was analyzed by IHC. Left panels show IHC staining of AXL and right panels show HE staining. Patient Nos. 1, 2 and 4 showed positive staining for AXL. Patient No. 5 showed negative staining for AXL (scale bar=50 μM). **(D)** Schema of small subpopulations of drug-tolerant tumor cells and novel therapeutic strategies.

The tumor volumes of xenograft mice implanted with H2228-CER cells were significantly reduced after treatment with ganetespib, or ganetespib in combination with ceritinib, after comparing tumor volumes at day 28 (Figure 5A). Ganetespib, in combination with ceritinib, reduced vimentin and ZEB1, and induced E-cadherin in western blot analyses of samples from mice after 28 days of treatment (Figure 5B). Ganetespib, in combination with ceritinib, also reversed EMT changes *in vivo*. These results suggest that a therapeutic approach that targets AXL may be expected to overcome drug-tolerant cancer cell subpopulations and have a profound impact on cancer eradication in ALK-positive NSCLC.

We finally investigated AXL protein expression, by IHC, in ALK-positive NSCLC patients treated with crizotinib as first line therapy and evaluated the correlation between AXL expression and crizotinib sensitivity. Seven ALK-positive NSCLC tissue samples from crizotinib treatment-naïve patients were available for analysis. Patient characteristics are shown in Supplementary Table 2. Figure 5C showed positive (No. 1, 2 and 4) and negative (No. 5) staining for AXL protein in ALK-positive NSCLC patient samples. All patients who were negative for AXL staining showed a partial response (PR) and long progression-free survival (PFS) to crizotinib. Cases Nos. 1 and 4 AXL-positive patients also displayed a long PFS with a complete response and a PR, respectively. However, a patient (No. 2) with positive AXL staining did not show a response to crizotinib. Another patient (No. 3) with positive AXL staining had a shorter PFS in response to crizotinib treatment. Although the sample size was small, the existence of drug-tolerant subpopulation with AXL overexpression before initial therapy may have also contributed to intrinsic resistance to ALK-TKIs in ALK-positive NSCLC patients.

## DISCUSSION

ALK signal–dependent activation, such as ALK secondary mutations, and the activation of alternative bypass signaling pathways, including *EGFR* and *KRAS,* are two major mechanisms of resistance to ALK-TKIs. The usefulness of alectinib and ceritinib for secondary mutations, and combination therapy such as EGFR-TKI has been shown previously [25–27]. However, it is still difficult to treat ALK-positive NSCLC because of the development of novel resistance mechanisms and a drug-tolerant cancer cell subpopulation. Sharma et al. consistently detected drug-tolerant cancer cell subpopulations in drug-sensitive human tumor cell lines that maintained viability under conditions where the vast majority of cells was rapidly killed after an acute response to various anti-cancer agents, including EGFR-TKI [28]. Hata et al. demonstrated that acquired resistance caused by the EGFR T790M mutation can occur in both pre-existing T790M clones and in the evolution of drug tolerant cells without T790M [29]. Late evolving T790M acquired resistance showed a decreased apoptotic response to EGFR-TKI. A previous study pointed to a likely relationship between drug-tolerant cancer cell and CSC subpopulations [30]. In addition, CSCs have been closely linked to the characteristics of EMT [31]. Therefore, the development of a fundamental method to overcome a drug-tolerant cell subpopulation and the origins of resistance, such as EMT and CSC, is critical for the elimination of cancer.

In this study, we established crizotinib, alectinib and ceritinib-resistant H2228 cells. These cells survived and showed a low proliferative ability that was commonly independent of ALK signaling. CSCs have been recognized as quiescent cells that are often identified by a lack of cell proliferation [32]. ALK-TKI-resistant NSCLC cells with CSC properties and an acquired EMT phenotype have been noted to be responsible for cancer survival. The EMT phenomenon is associated with drug resistance, including EGFR-TKI and ALK-TKI [31, 33–35]. AXL activation and MED12 loss induced an EMT-like phenotype, which is associated with EGFR-TKI resistance in lung cancer [33, 36]. We have previously reported that TGF-β1-induced EMT changes in NSCLC cells with drug insensitivity were associated with low expression of the miR-200 family and the alteration of EMT-related factors such as ZEB1 and ZEB2 [19, 37]. In this study, we observed AXL-driven EMT changes in all established, ALK signal-independent ALK-TKI-resistant cells. AXL overexpression resulting in EMT was also found in H2228 cells after exposure to TGF-β1. Although further clinical data is needed, AXL protein overexpression is suggested to be associated with a poor response to first-line crizotinib therapy in ALK-positive NSCLC patients.

AXL is classified as a receptor tyrosine kinase belonging to the TAM subfamily [38]. We recently reported the prognostic significance of the co-expression of AXL and its ligand, GAS6, in lung adenocarcinoma [39]. AXL activation and EMT features have also been reported as mechanisms of resistance to targeted therapy in human cancers, including NSCLC [20, 33, 40]. We observed that EMT induced in response to AXL overexpression contributed to intrinsic as well as acquired resistance to ALK-TKIs. Debruyne et al. have reported that the ALK point mutations of the kinase domain (F1174L) induced AXL activation, EMT and ALK-TKI-resistance in neuroblastoma models [41]. The different ALK-TKI-resistant model used was derived from a different type of cancer and appeared to be similar to that of our study An AXL inhibitor is a promising drug in current development to treat human cancer [42–44]. BGB324 (R428) is a selective AXL inhibitor and has been used for phase I studies in patients with refractory/relapsed acute myeloid leukemia (AML) and high-risk myelodysplastic syndromes [43]. Gilteritinib (ASP2215) is a highly potent inhibitor of FLT3 and AXL, showing antileukemic activity against AML with either or both FLT3-ITD and FLT3-D835 mutations [44]. In the current study, the inhibition of AXL was effective for all three ALK-TKI–resistant cell lines. Thus, AXL associated with the development of EMT may be a promising therapeutic target for the elimination of cancer in ALK-positive NSCLC cells.

We found that an HSP90 as well as an AXL inhibitor could reverse TGF-β1-induced EMT in H2228 cells and was also effective in ALK-TKI-resistant H2228 cells. HSP90 exhibits protective chaperone properties through its protein folding activity, with client proteins that include AXL, mutant EGFR and ALK [45]. H2228 cells express EML4-ALK fusion protein variants 3a and 3b, which are not client proteins for HSP90 as they lack the TAPE domain of EML4 [46]. Therefore, any effect of ganetespib as described in our manuscript should be regarded as an ALK independent effect on EMT. Previous studies reported that HSP90 inhibitors induced sensitivity in crizotinib-resistant NSCLC patients with a secondary ALK mutation, as well as in crizotinib-resistant H2228 cells [22]. In a phase II study of NSCLC tumors, all four patients showing a partial response had an *ALK* gene rearrangement [24]. An HSP90 inhibitor may be a promising drug against ALK-TKI-resistant NSCLC, with or without ALK signaling.

Our study had an important limitation. The significance of ALK signaling-independent AXL overexpression associated with a drug-tolerant subpopulation and commonly involved in clinical alectinib, crizotinib and ceritinib resistance was observed in only one cell line. A further study will be performed to confirm this significance using another ALK-positive NSCLC cell line.

In conclusion, we found that ALK signaling-independent AXL overexpression associated with EMT features and CSC properties was commonly involved in the maintenance of cancer survival, and was critical in intrinsic and acquired resistance to first-and second-generation ALK-TKIs. AXL and HSP90 inhibitors may be effective against a drug-tolerant cancer cell subpopulation that has caused relapse and drug resistance in patients. Therefore, AXL inhibition using AXL and HSP90 inhibitors may be a promising therapeutic strategy in ALK-positive NSCLC patients in both initial and recurrent treatment phases. Such inhibitors may also be useful in dealing a final blow to the disease after using front-line ALK-TKIs in ALK-positive NSCLC patients (Figure 5D). Further studies should be performed on the significance of AXL and HSP90 inhibitors in a drug-tolerant cancer cell subpopulation.”

## MATERIALS AND METHODS

### Cell culture

H2228 (*EML4-ALK* variant 3a/b E6; A20), a human non–small cell lung cancer cell line, was obtained from the American Type Culture Collection in 2013. Cells were amplified and frozen, and one aliquot was thawed for this study. All cells were routinely screened for the absence of mycoplasma and maintained in RMPI 1640 medium (Sigma–Aldrich, St. Louis, MO) with 10% heat-inactivated fetal bovine serum (FBS) and 1% penicillin/streptomycin at 37°C in a 5% CO_2_ incubator.

### Drugs and cell viability assay

The ALK inhibitors, crizotinib, alectinib (CH5424802) and ceritinib (LDK378), the AXL inhibitor, R428, and the HSP90 inhibitor, ganetespib, were obtained from Selleck Chemicals (Houston, TX). TGF-β1 was purchased from R and D Systems (Minneapolis, MN). To evaluate their sensitivity to drugs *in vitro*, H2228 cells were seeded (2,000 cells/well) in 96-well plates (PrimeSurface MS-9096U, Sumitomo Bakelite Co., Ltd., Tokyo, Japan) and incubated at 37°C for 24 hours. Cells were then incubated with various concentrations of compounds or vehicle (DMSO) at 37°C for 72 hours. Viability experiments were performed using a CellTiter-Glo Luminescent Cell Viability Assay (Promega Corporation, Madison, WI) and microplate reader (Infinite M200 PRO; Tecan Group Ltd, Männedorf, Switzerland) according to the manufacturer’s instructions. Results were plotted as nonlinear regression curves and IC_50_ values calculated using GraphPad Prism 7 software (La Jolla, CA, USA). We used a SCADS Inhibitor Kit III, which included 95 chemical compounds that each inhibited a specific molecular target, as previously described [21].

### Fluorescence *in situ* hybridization

Break-apart fluorescence *in situ* hybridization (FISH) analysis was performed with a Vysis ALK break-apart FISH probe (Abbott Molecular, Des Plaines, IL). The 3′ (red) and 5′ (green) signals separated by ≥ 2 signal diameters were considered split as positive.

### Western blot analysis and receptor tyrosine kinase phosphorylation antibody arrays

Protein extraction and western blot analyses were performed as previously described [19, 47]. Antibodies to ALK, p-ALK, AXL, vimentin, ZEB1, p-EGFR, EGFR, AKT, p-AKT, Erk1/2 and p-Erk1/2 were obtained from Cell Signaling Technology (Danvers, MA). An antibody to E-cadherin was purchased from Santa Cruz Biotechnologies (Santa Cruz, CA). An antibody to p-AXL was obtained from R and D Systems. An antibody to β-actin was purchased from Sigma–Aldrich (St. Louis, MO). We performed human receptor tyrosine kinase (RTK) phosphorylation antibody analysis using RayBio Human RTK Phosphorylation Antibody Array G-series 1, which included 71 antibodies (RayBiotech, Inc., Norcross, GA).

### RNA extraction and microarray analysis

Total RNA from cells was extracted using TRIzol Reagent (Thermo Fisher Scientific, Waltham, MA) as previously described [48, 49], or an RNeasy Plus Mini kit (QIAGEN, Hilden, Germany). Gene expression microarray analysis was carried out using a GeneChip Human Gene 2.0 Sense Target array (Affymetrix, Santa Clara, CA) according to the manufacturer’s protocol. We also performed miRNA analysis using 3D-Gene Human miRNA oligo chips ver.21 (TORAY, Tokyo, Japan). Microarray data have been deposited in NCBI’s Gene Expression Omnibus (GEO; http://www.ncbi.nlm.nih.gov/geo/) and are accessible through a GEO series accession number GSE94809.

### Quantitative real-time reverse transcription–PCR

For quantitative real-time reverse transcription–PCR (qRT-PCR), expression levels of miRNAs were measured using a Taqman MicroRNA Assay (Thermo Fisher Scientific) and a 7500 fast real-time PCR system. The *RNU66* expression level was used as an internal control. The gene expression of cancer stem cell–related genes, including *ALDH1A*, *ABCB1* and *ABCG2*, was examined by TaqMan Gene Expression Assay (Thermo Fisher Scientific). Expression levels of miRNAs and genes were quantified as 2^-ΔΔCt^ values [50].

### Exome sequencing

DNA was extracted from cultured cell lines using a QIAamp DNA mini kit (QIAGEN, Hilden, Germany) according to the manufacturer’s protocol. Exome sequencing of extracted DNA was conducted on an Illumina HiSeq 2500 platform using paired-end reads (Illumina, San Diego, CA). Reads were aligned against the reference human genome and compared with each cell line.

### Sphere formation assay

Cells (2,000 cells per well) were plated in 24-well ultralow attachment cell culture plates (Corning Inc., Corning, NY) and cultured in serum-free DMEM/F-12 medium (Thermo Fisher Scientific) with 20 ng/mL epidermal growth factor (EGF; Thermo Fisher Scientific), 20 ng/mL basic fibroblast growth factor (bFGF) (ReproCELL, Yokohama, Japan), B27 (Thermo Fisher Scientific), heparin (STEMCELL Technologies, Vancouver, Canada) and 0.03% LA-717 (SphereMax, Nissan Chemical Industries, Ltd., Tokyo, Japan). The ability to form spheres was determined by counting the number of spheres with a diameter more than 100 μm after 7 days of culturing.

### RNA interference

Gene knockdown of AXL was performed by transfection using Lipofectamine RNAiMAX (Thermo Fisher Scientific) and ON-TARGETplus AXL siRNA Reagent (Dharmacon, Inc., Lafayette, CO). An ON-TARGETplus Non-targeting Control Pool (Dharmacon) was used as a negative control.

### Tumor cell implantation experiments

Female severe combined immunodeficient (SCID)-Beige mice (CB17.Cg-Prkdc^scid^Lyst^bg-J^/CrlCrlj) were purchased at 5 weeks of age (Charles River Laboratories Japan Inc., Yokohama, Japan). H2228 or H2228-CER cells (1 × 10^7^) were injected subcutaneously into the underarms of 6-week-old mice. We performed experiments to confirm the transforming potential and response to drugs of ALK-TKI–resistant cells. In the response experiment to drugs, mice were randomized (day 3) to the following four cohorts (n=6, for each group): vehicle control (per os (p.o.), once a day), ceritinib (p.o., dosed with 25 mg/kg, once a day), ganetespib (intravenous (i.v.), dosed with 50 mg/kg, once a week) or both ceritinib (p.o., dosed with 25 mg/kg, once a day) and ganetespib (i.v., dosed with 50 mg/kg, once a week) were administrated until the end of the 28-day treatment period. Body weights were measured once a week. Tumor volumes (V) were calculated twice a week by caliper measurements of the width (W) and length (L) of each tumor (W^2^×L/2). Statistical analysis was performed based measurements on day 28. Protein extraction and western blot analyses were performed using samples from mice after the 28-day treatment period.

### Immunohistochemical analysis

Seven tumor specimens with *ALK* gene translocation were obtained from seven lung adenocarcinoma patients, all of whom had received crizotinib, at Nippon Medical School Hospital between 2006 and 2016. Tumor samples were obtained from resections and transbronchial lung biopsies. Patients’ characteristics are shown in Supplementary Table 2. The response to crizotinib was evaluated by Response Evaluation Criteria in Solid Tumors (RECIST) version 1.0. For immunohistochemical (IHC) staining of AXL, formalin-fixed paraffin-embedded tissue sections were stained by an immunoperoxidase method as previously described [51, 52]. Slides were incubated with a primary antibody against AXL obtained from R and D Systems. Positive staining was defined as membranous and/or cytoplasmic staining in greater than 10% of tumor cells. We also performed Hematoxylin-eosin (HE) staining. The study protocol was approved by an ethics committee review board at Nippon Medical School Hospital. Written, informed consent was obtained from all patients, and patient specimens were collected in accordance with the Declaration of Helsinki 2013.

### Statistical analysis

Data were expressed as the mean ± standard error (SE) of three independent experiments and evaluated with Student’s *t* test. A *P* value of < 0.05 was considered to be significant.

## SUPPLEMENTARY MATERIALS FIGURES AND TABLES




