# Supplementary material for: Overcoming drug-tolerant cancer cell subpopulations showing AXL activation and epithelial–mesenchymal transition is critical in conquering ALK-positive lung cancer
Source: Oncotarget. 2018 Jun 5;9(43):27242–55. doi: 10.18632/oncotarget.25531 (PMC6007478; doi:10.18632/oncotarget.25531)
Supplement: Supplementary file 1 [file oncotarget-09-27242-s001.pdf]

Overcoming drug-tolerant cancer cell subpopulations showing AXL activation and epithelial–mesenchymal transition is critical in conquering ALK-positive lung cancer

SUPPLEMENTARY MATERIALS

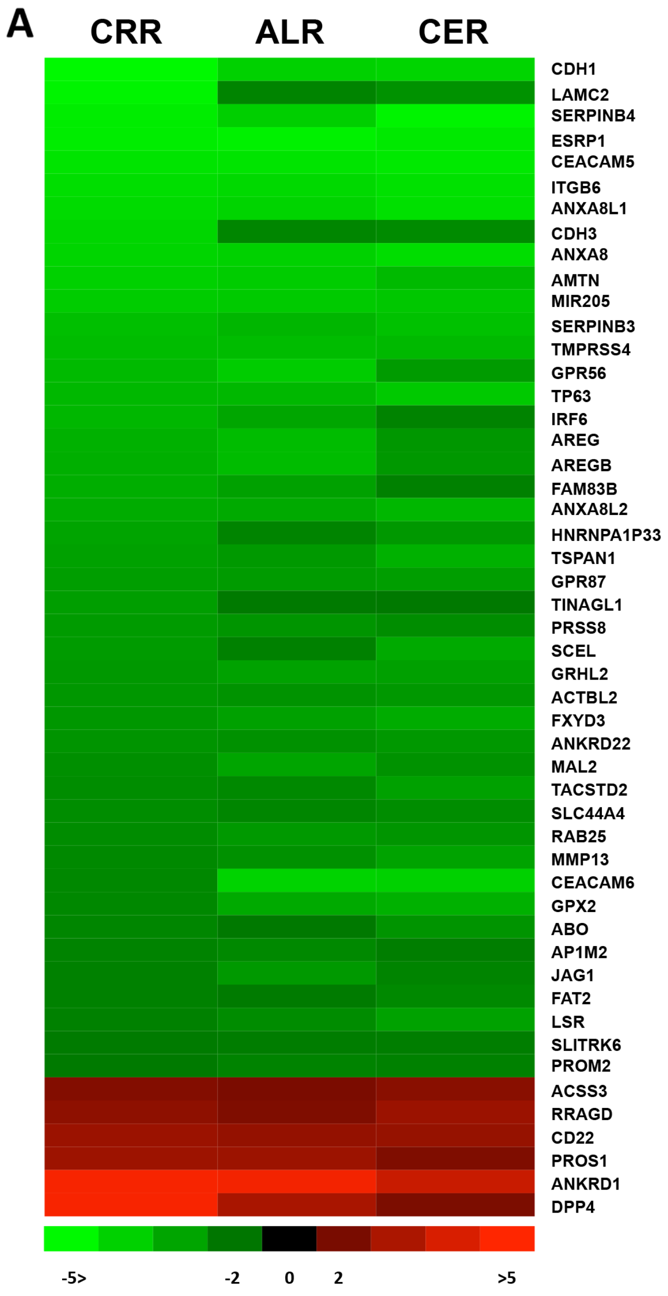

Supplementary Figure 1A: The cDNA microarrays undertaken in ALK-TKI–resistant cells compared with parental cells are shown by a heat map using $\log_2$ ratio [$\log_2$(ALK-TKI–resistant H2228 cells/H2228 cells)] (common $\log_2$ ratio changes of >5 or <-5).

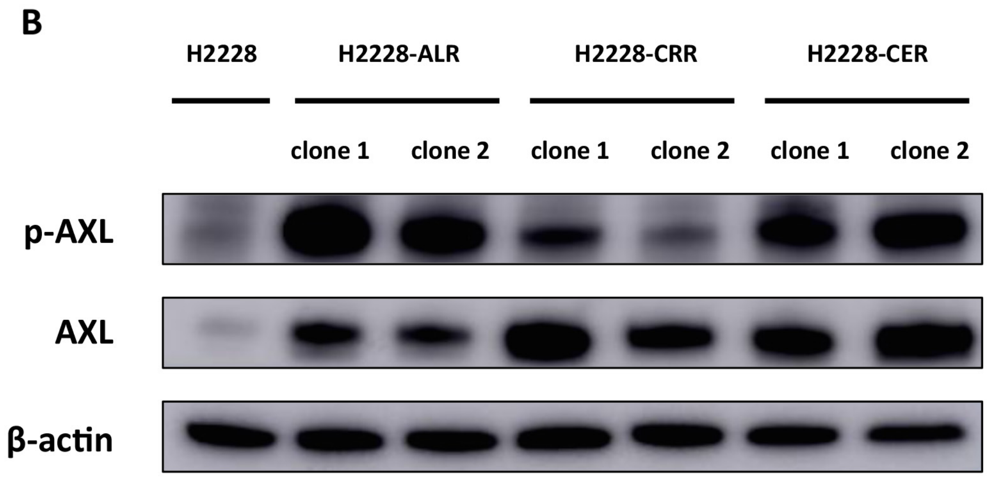

Supplementary Figure 1B: Protein levels of p-AXL and AXL were examined by western blot analysis in two independent clones of each ALK-TKI-resistant cell line. Upregulated AXL was also confirmed in cloned ALK-TKI–resistant H2228 cells.

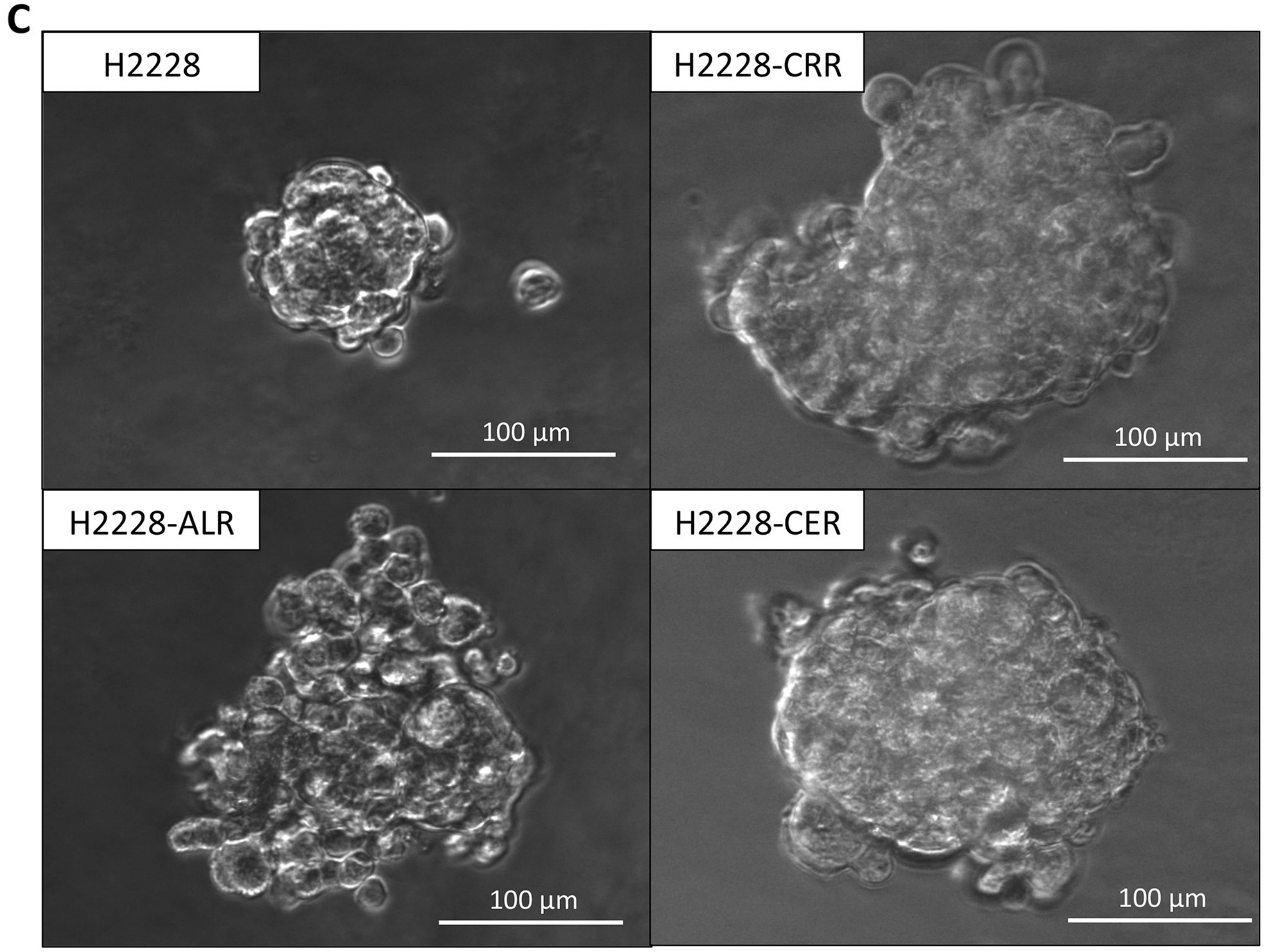

Supplementary Figure 1C: ALK-TKI–resistant H2228 cells were able to form large spheres compared to parental H2228 cells in the sphere formation assay.

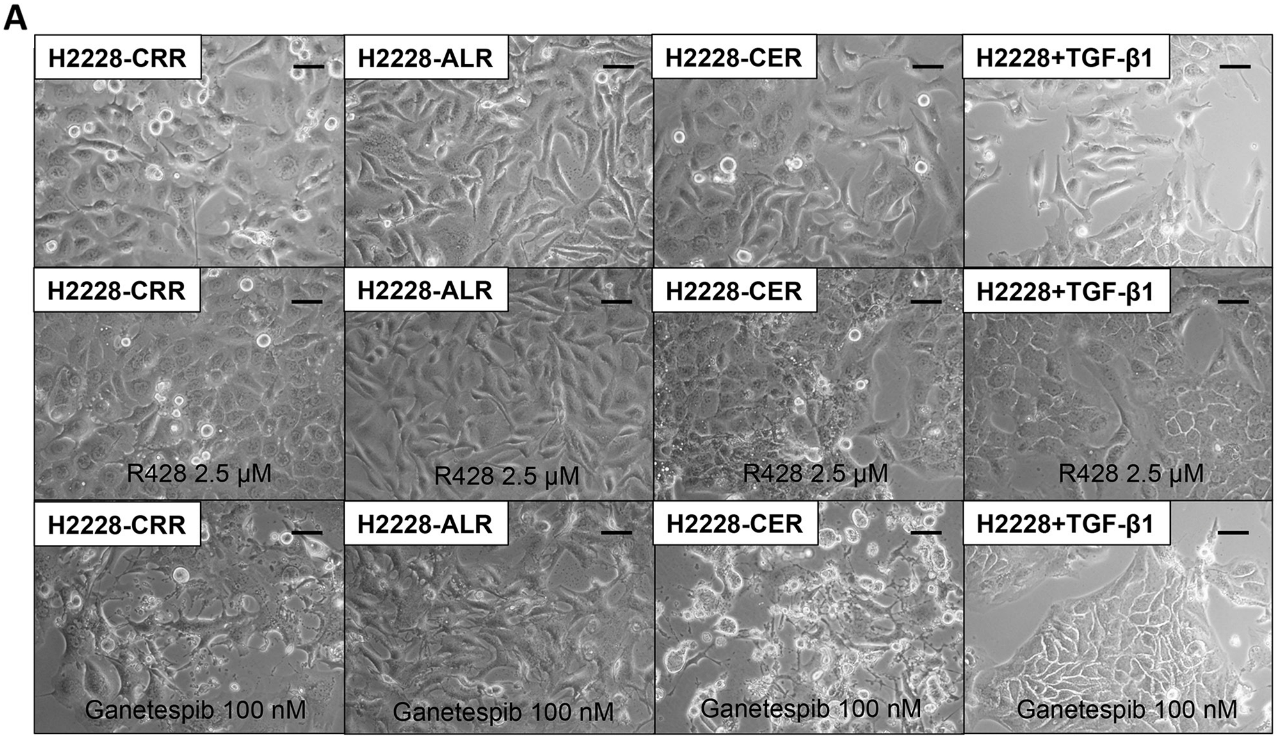

Supplementary Figure 2A: The disappearance of mesenchymal features in three ALK-TKI–resistant cell lines (-CRR, -ALR, -CER) and in TGF-β1 treated H2228 cells was observed 48 hours after treatment with 2.5 µM R428 or 100 nM ganetespib (scale bar=50 µM).

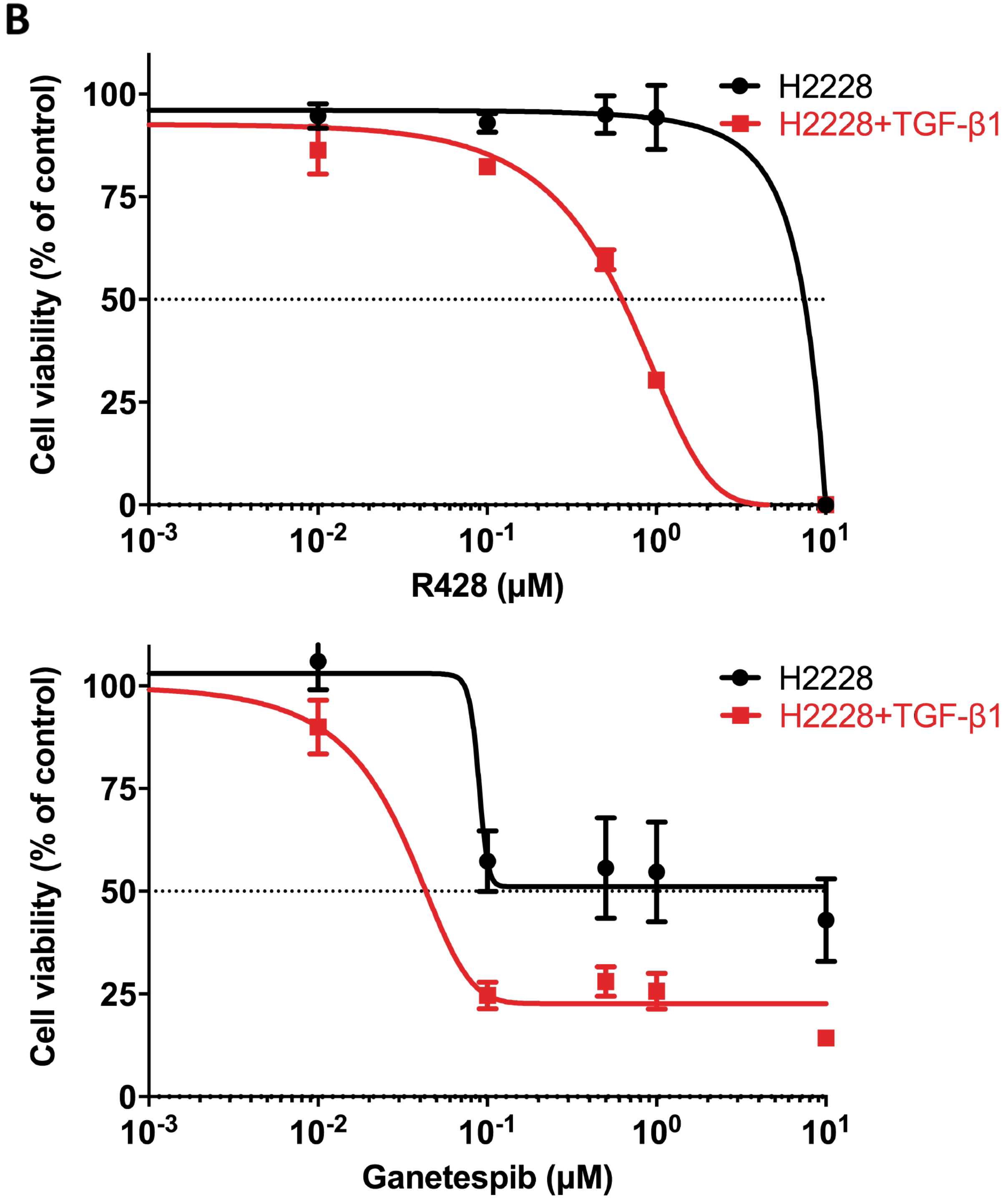

Supplementary Figure 2B: The effectiveness of R428 and ganetespib in TGF-β1 treated H2228 cells compared with untreated H2228 cells as shown by cell viability assay.

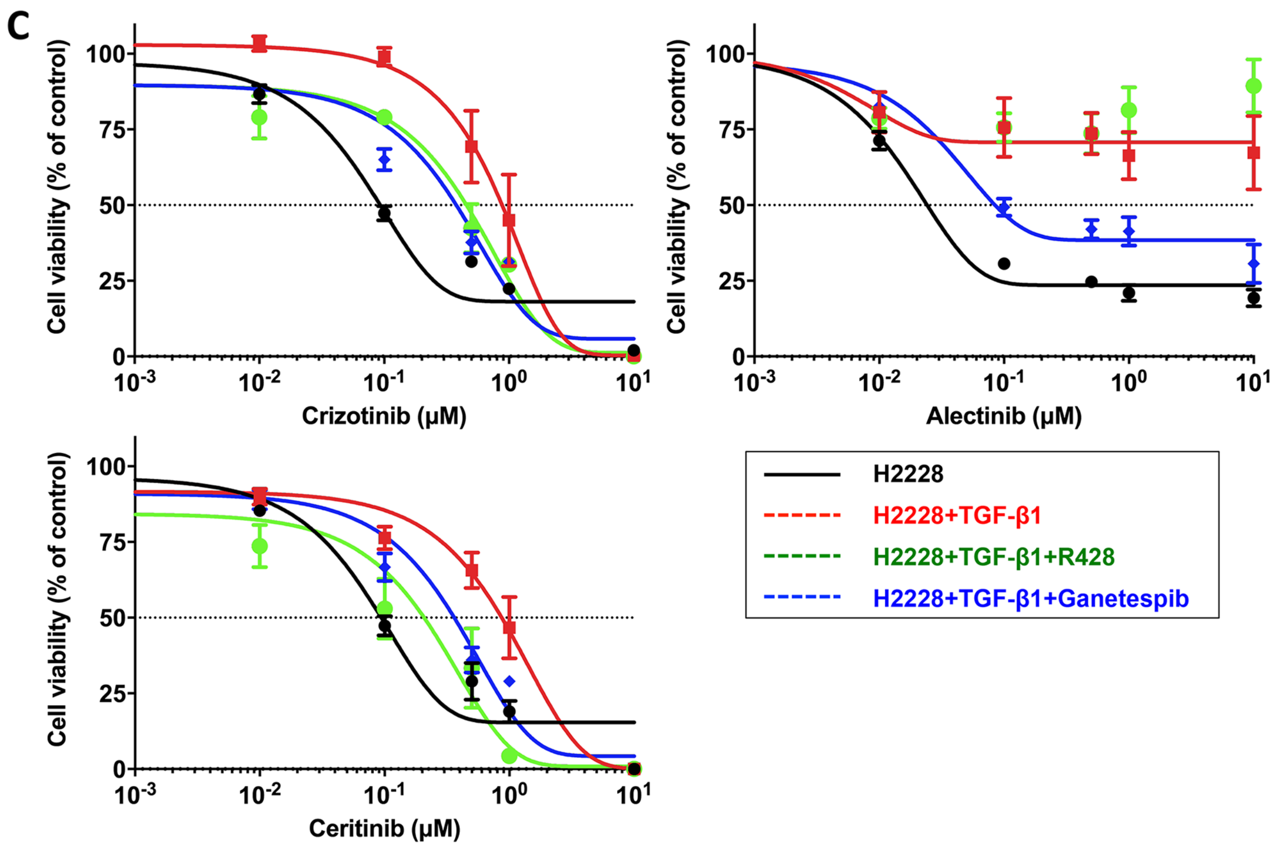

Supplementary Figure 2C: Reversal of the resistance to ALK-TKIs in TGF-β1 treated H2228 cells by R428 or ganetespib as shown by cell viability assay.

Supplementary Table 1: Screening analysis using 95 chemical compounds at 500 nM to identify candidate molecules for ALK-TKI–resistant cells using a SCADS Inhibitor Kit III.

See Supplementary File 1

Supplementary Table 2: Characteristics of ALK-positive NSCLC patients treated with crizotinib as first line therapy and results of AXL staining

Patient No.	Age	Gender	AXL staining	Crizotinib Response (RECIST)	PFS (months)
1	65	M	Positive	CR	28.7
2	73	F	Positive	PD	0.2
3	36	M	Positive	PR	7.5
4	28	F	Positive	PR	22.5
5	52	M	Negative	PR	20.2
6	74	M	Negative	PR	12.2
7	73	M	Negative	PR	11.5

Abbreviations: M, male; F, female; PFS, progression-free survival; PR, partial response; CR, complete response; PD, progression of disease; RECIST, response evaluation criteria in solid tumors.
