# Supplementary material for: Overcoming drug-tolerant cancer cell subpopulations showing AXL activation and epithelial–mesenchymal transition is critical in conquering ALK-positive lung cancer
Source: Oncotarget. 2018 Jun 5;9(43):27242–55. doi: 10.18632/oncotarget.25531 (PMC6007478; doi:10.18632/oncotarget.25531)
Supplement: Supplementary file 2 [file oncotarget-09-27242-s002.docx]

**Supplementary Table 1.** **Screening analysis using 95 chemical compounds at 500 nM to identify candidate molecules for ALK-TKI–resistant cells using a SCADS Inhibitor Kit III.**

| Category | Compound | IC50 (µM)[target] | Cell viability | | | |
| --- | --- | --- | --- | --- | --- | --- |
|  |  |  | H2228 | CRR | ALR | CER |
| Hsp90 | radicicol | 0.27 nM [v-src] | 0.62 | 0.86 | 0.59 | 0.89 |
| ATM | ATM/ATR kinase inhibitor | 0.2 [ATM] | 0.84 | 1.10 | 0.86 | 0.81 |
| CDK | Cdk2/9 inhibitor | 0.002 [Cdk2], 0.004 [Cdk9] | 0.51 | 0.56 | 0.98 | 0.81 |
| PDGFR | SU11652 | 0.003 [PDGFR], 0.027 [VEGFR2], 0.17 [VEGFR1] | 0.63 | 1.01 | 0.85 | 0.81 |
| Raf | RAF1 kinase inhibitor I | 0.009 [Raf] | 0.84 | 0.74 | 0.85 | 0.81 |
| CDK | Cdk1/2 inhibitor III | 600pM [Cdk1], 500 pM [Cdk2] | 0.79 | 0.79 | 0.61 | 0.82 |
| CAMKII | Lavendustin C | 0.2 [CAMKII], 0.2 [c-src] | 0.75 | 1.12 | 1.00 | 0.83 |
| PKG | Rp-8-CPT-cGMPS |  | 0.84 | 1.14 | 0.90 | 0.83 |
| ROCK | H-1152 | 0.002 [ROCK] | 1.01 | 0.68 | 0.90 | 0.83 |
| TGF-βRI | SB431542 |  | 1.23 | 1.25 | 1.04 | 0.83 |
| EGFR | BPIQ-Ⅱ | 8 pM [EGFR] | 0.86 | 0.94 | 0.88 | 0.86 |
| Jak | JAK Inhibitor I | 0.015 [JAK1], 0.001 [JAK2], 0.005 (Ki) [JAK3], 0.001 [Tyk2] | 0.82 | 0.62 | 0.92 | 0.86 |
| GSK | indirubin-3'-monoxime | 0.022 [GSK-3b], 0.18 [Cdk1], 0.1 [Cdk5] | 0.75 | 0.86 | 0.95 | 0.87 |
| Lck | PP2 | 0.004 [Lck] | 0.73 | 0.85 | 0.92 | 0.88 |
| PKA | H-89 |  | 1.16 | 1.05 | 0.99 | 0.88 |
| CK | TBB | 0.9 [CKII] | 1.00 | 0.92 | 0.95 | 0.90 |
| MEK | U-0126 | 0.072 [MEK1], 0.058 [MEK2] | 0.83 | 0.96 | 0.90 | 0.90 |
| DNA-PK | IC60211 | 0.43 [DNA-PK] | 1.02 | 1.01 | 0.99 | 0.92 |
| JNK | JNK inhibitor VIII | 0.002 [JNK] | 0.87 | 1.02 | 1.09 | 0.92 |
| PKC | Go7874 | 0.004 [PKC] | 0.86 | 0.85 | 0.86 | 0.92 |
| Fms | cFMS Receptor Tyrosine Kinase Inhibitor | 0.03 [cFMS] | 0.98 | 1.08 | 0.86 | 0.93 |
| IKK | IKK-2 inhibitor VI | 0.013 [IKK-2] | 1.05 | 0.98 | 0.93 | 0.93 |
| Raf | ZM 336372 | 0.07 [Raf] | 0.98 | 0.99 | 1.00 | 0.93 |
| VEGFR | VEGF receptor 2 kinase inhibitor I | 0.07 [VEGFR2] | 0.89 | 0.77 | 0.93 | 0.93 |
| AK | ABT-702 | 0.05 [AK] | 1.13 | 0.97 | 1.13 | 0.94 |
| CAMKII | KN-93 | 0.37 (Ki) [CAMKII] | 1.04 | 0.98 | 0.98 | 0.94 |
| CK | Ellagic acid | 0.04 [CK2] | 0.94 | 1.08 | 0.95 | 0.94 |
| Aurora | Aurora kinase inhibitor II | 0.31 [aurora-A], 0.24 [aurora-B] | 1.24 | 1.02 | 0.96 | 0.95 |
| BTK | LFM-A13 | 2.5 [BTK] | 0.83 | 0.90 | 1.00 | 0.95 |
| CDK | NU6102 | 0.01 [Cdk1], 0.005 [Cdk2] | 1.91 | 1.69 | 1.20 | 0.95 |
| Fyn | SU6656 | 0.17 [Fyn], 0.28 [Src] | 0.90 | 0.97 | 1.06 | 0.95 |
| MEK | MEK inhibitor I | 0.012 [MEK] | 0.91 | 1.03 | 1.00 | 0.95 |
| p38 | SB202190 | 0.016 [p38b] | 0.83 | 0.91 | 1.00 | 0.95 |
| VEGFR | SU1498 | 0.7 [Flk-1] | 1.17 | 0.75 | 1.09 | 0.95 |
| ATM | ATM kinase inhibitor | 0.013 [ATM] | 0.92 | 0.79 | 1.05 | 0.96 |
| CDK | Olomoucine |  | 0.90 | 0.92 | 1.03 | 0.96 |
| Bcr-abl | AG957 | 0.75 (Ki) [Bcr-abl] | 1.04 | 1.05 | 1.04 | 0.97 |
| PDGFR | PDGF receptor tyrosine kinase inhibitor V | 0.004 [PDGFR] | 1.22 | 1.00 | 0.99 | 0.97 |
| Tpl2 | Tpl2 kinase inhibitor | 0.05 [Tpl2] | 0.82 | 1.03 | 0.91 | 0.97 |
| AKT | Akt Inhibitor VIII, Isozyme-Selective, Akti-1/2 | 0.058 [Akt1], 0.21 [Akt2], 2.1 [Akt3] | 0.94 | 1.06 | 1.00 | 0.98 |
| CDK | Cdk4 inhibitor | 0.076 [Cdk4] | 1.00 | 0.92 | 0.81 | 0.98 |
| Chk | SB218078 |  | 0.71 | 0.67 | 0.88 | 0.98 |
| IGF-IR | AGL 2263 | 0.4 [IR], 0.43 [IGF-1R] | 0.93 | 1.00 | 1.01 | 0.98 |
| PDGFR | PDGF receptor tyrosine kinase inhibitor IV | 0.004 [PDGFR] | 0.73 | 0.83 | 1.14 | 0.98 |
| CAMKII | KN-62 | 0.9 [CAMKII] | 1.14 | 0.94 | 1.02 | 0.99 |
| Chk | Chk2 inhibitor | 0.008 [Chk2] | 1.07 | 1.14 | 0.99 | 0.99 |
| eEF2 | TX-1918 | 0.44 [eEF2-K] | 0.88 | 0.97 | 0.89 | 1.00 |
| EGFR | AG1478 | 0.003 [EGFR] | 1.15 | 0.91 | 0.93 | 1.00 |
| GSK | GSK-3 inhibitor IX | 0.005 [GSK-3a] | 1.04 | 1.02 | 1.03 | 1.00 |
| IKK | BMS-345541 | 0.3 [IKK-2] | 1.03 | 0.92 | 1.00 | 1.00 |
| CK | DMAT | 0.14 [CK2] | 0.95 | 0.98 | 1.12 | 1.01 |
| MEK | PD98059 | MEK | 1.22 | 0.92 | 0.98 | 1.01 |
| TrKA | TrkA inhibitor | 0.006 [TrkA] | 1.05 | 1.03 | 0.94 | 1.01 |
| BTK | Terreic acid | 3 [BTK] | 0.98 | 1.05 | 0.95 | 1.02 |
| FGFR | SU4984 |  | 0.89 | 0.98 | 1.06 | 1.02 |
| Lck | Damnacanthal | p56lck | 0.92 | 1.06 | 1.10 | 1.02 |
| TGF-βRI | TGF-b RI kinase inhibitor II | 0.023 [TGF-bRI] | 1.09 | 1.08 | 1.03 | 1.02 |
| Flt-3 | Flt-3 Inhibitor | 0.042 [Flt-3] | 1.07 | 0.95 | 1.07 | 1.03 |
| PKC | Bisindolylmaleimide I, HCl | 0.01 [PKC] | 1.29 | 0.96 | 1.01 | 1.03 |
| PKG | KT5823 | 0.23 [PKG] | 0.89 | 0.77 | 1.09 | 1.03 |
| DGK | Diacylglycerol kinase inhibitor II | 0.12 [DGK] | 1.05 | 0.92 | 1.27 | 1.04 |
| GSK | 1-Azakenpaullone | 0.018 [GSK-3b] | 1.17 | 0.96 | 0.86 | 1.04 |
| JNK | SP600125 | 0.04 [JNK] | 1.27 | 1.08 | 0.86 | 1.04 |
| Chk | Chk2 inhibitor II | 0.015 [Chk2] | 0.97 | 0.89 | 0.98 | 1.05 |
| CK | D4476 | 0.2 [CK1] | 1.22 | 1.07 | 0.97 | 1.05 |
| PKA | 4-cyano-3-methylisoquinoline | 0.03 [PKA] | 0.88 | 1.21 | 1.05 | 1.05 |
| IRAK | IRAK-1/4 inhibitor | 0.3 [IRAK] | 0.97 | 1.06 | 0.98 | 1.06 |
| p38 | SB239063 | 0.044 [p38a, b] | 0.98 | 1.03 | 0.97 | 1.06 |
| VEGFR | VEGFR receptor tyrosine kinase inhibitor II | 0.02 [KDR], 0.18 [Flt-1], 0.24 [c-Kit] | 0.89 | 0.95 | 1.03 | 1.06 |
| FGFR | SU5402 |  | 0.94 | 1.02 | 1.02 | 1.08 |
| PDGFR | AG1296 | 1 [PDGFRa], 0.8 [PEGFRb] | 1.25 | 0.95 | 0.99 | 1.08 |
| Aurora | Aurora kinase/cdk inhibitor | 0.011 [aurora-A], 0.015 [aurora-B] | 1.20 | 1.16 | 0.95 | 1.09 |
| HER2 | AG825 | 0.35 [HER2], 19 [EGFR] | 1.37 | 1.14 | 1.08 | 1.09 |
| Met | SU11274 | 0.02 [Met] | 1.14 | 1.11 | 1.02 | 1.09 |
| EGFR | AG490 | JAK-2 | 1.25 | 1.24 | 1.16 | 1.10 |
| PI3K | LY-294002 | 1.4 [PI3K] | 1.46 | 1.19 | 1.07 | 1.10 |
| CDK | NSC625987 | 0.2 [Cdk4] | 1.27 | 1.24 | 1.18 | 1.11 |
| IGF-IR | AG1024 | IGF-IR, IR | 0.96 | 1.07 | 1.11 | 1.12 |
| Jak | JAK3 Inhibitor VI | 0.027 [JAK3] | 1.15 | 1.41 | 1.28 | 1.12 |
| ROCK | Y-27632 | 0.14 [ROCK] | 1.08 | 1.14 | 1.07 | 1.12 |
| Src | PP1 analog | 1.5 nM [v-src] | 1.34 | 1.22 | 1.64 | 1.12 |
| AKT | Akt Inhibitor XI | 0.1 [Akt] | 1.17 | 1.28 | 1.19 | 1.13 |
| Chk | isogranulatimide | 0.1 [Chk1] | 1.18 | 1.46 | 1.31 | 1.13 |
| Clk | TG003 | 0.01 [Clk1] | 0.95 | 1.08 | 0.97 | 1.13 |
| PI3K | Wortmannin |  | 1.05 | 0.95 | 0.99 | 1.13 |
| AKT | Akt Inhibitor IV |  | 0.95 | 0.83 | 0.89 | 1.15 |
| AMPK | compound C | 0.1 [AMPK] | 1.07 | 1.07 | 1.01 | 1.16 |
| CDK | purvalanol A | 0.004 [cdc2], 0.07 [cdk2/A], 0.035 [cdk2/E], 0.075 [cdk5] | 0.95 | 0.90 | 0.96 | 1.16 |
| Aurora | Aurora kinase inhibitor III | 0.042 [aurora-A] | 1.04 | 0.98 | 1.13 | 1.17 |
| MAPK | ERK inhibitor II | 0.51 [ERK1], 0.33 [ERK2] | 1.33 | 1.17 | 1.00 | 1.17 |
| CDK | Alsterpaullone, 2-cyanoethyl | 230pM [Cdk1 ] | 1.29 | 1.30 | 1.22 | 1.18 |
| Syk | Syk inhibitor | 0.014 [Syk] | 1.07 | 0.90 | 1.06 | 1.18 |
| MLCK | ML-7 | 0.3 [MLCK] | 1.04 | 0.93 | 1.08 | 1.25 |
| CDK | Kenpaullone | CDK1/cycB | 1.09 | 1.01 | 1.13 | 1.27 |
| PKR | PKR inhibitor | 0.21 [PKR] | 1.46 | 1.78 | 1.28 | 1.53 |
